# Serum Proteome Signatures of Anti-SARS-CoV-2 Vaccinated Healthcare Workers in Greece Associated with Their Prior Infection Status †

**DOI:** 10.3390/ijms231710153

**Published:** 2022-09-05

**Authors:** Eleni Stamoula, Eleana Sarantidi, Vasilis Dimakopoulos, Alexandra Ainatzoglou, Ioannis Dardalas, Georgios Papazisis, Konstantina Kontopoulou, Athanasios K. Anagnostopoulos

**Affiliations:** 1 Department of Biotechnology, Centre of Systems Biology, Biomedical Research Foundation of the Academy of Athens, 11527 Athens, Greece; 2 Department of Clinical Pharmacology, School of Medicine, Aristotle University of Thessaloniki, 54636 Thessaloniki, Greece; 3 Clinical Research Unit, Special Unit for Biomedical Research and Education, School of Medicine, Aristotle University of Thessaloniki, 54636 Thessaloniki, Greece; 4 Laboratory of Microbiology, “G. Gennimatas” General Hospital, 54635 Thessaloniki, Greece

**Keywords:** COVID-19, SARS-CoV-2, vaccination, proteomics, serum, LC-MS/MS

## Abstract

Over the course of the pandemic, proteomics, being in the frontline of anti-COVID-19 research, has massively contributed to the investigation of molecular pathogenic properties of the virus. However, data on the proteome on anti-SARS-CoV-2 vaccinated individuals remain scarce. This study aimed to identify the serum proteome characteristics of anti-SARS-CoV-2 vaccinated individuals who had previously contracted the virus and comparatively assess them against those of virus-naïve vaccine recipients. Blood samples of *n* = 252 individuals, out of whom *n* = 35 had been previously infected, were collected in the “G. Gennimatas” General Hospital of Thessaloniki, from 4 January 2021 to 31 August 2021. All participants received the BNT162b2 mRNA COVID-19 vaccine (Pfizer/BioNTech). A label-free quantitative proteomics LC-MS/MS approach was undertaken, and the identified proteins were analyzed using the GO (Gene Ontology) and KEGG (Kyoto Encyclopedia of Genes) databases as well as processed by bioinformatics tools. Titers of total RBD-specific IgGs against SARS-CoV-2 were also determined using the SARS-CoV-2 IgG II Quant assay. A total of 47 proteins were significantly differentially expressed, the majority of which were down-regulated in sera of previously infected patients compared to virus-naïve controls. Several pathways were affected supporting the crucial role of the humoral immune response in the protection against SARS-CoV-2 infection provided by COVID-19 vaccination. Overall, our comprehensive proteome profiling analysis contributes novel knowledge of the mechanisms of immune response induced by anti-SARS-CoV-2 vaccination and identified protein signatures reflecting the immune status of vaccine recipients.

## 1. Introduction

Coronavirus disease 2019 (COVID-19) has been an important global viral health threat for the last two and a half years. It is characterized by very high transmissibility and low to high severity depending on patient age and underlying comorbidities. The impact of the pandemic in public health, social care systems and the economy of countries has been devastating. Most patients with COVID-19 will recover, some will die, and many will develop long-COVID, a long lasting state with symptoms like fatigue, shortness of breath, arthralgia, myalgia, insomnia, cardiac abnormalities, cognitive impairment, symptoms of post-traumatic stress disorder, concentration problems, and headaches [1,2,3]. To date, no effective treatment is available, while vaccines, despite being highly effective in reducing patient hospitalization and mortality, are not sufficient in completely eliminating the disease and its repercussions. Thus, understanding the pathogenesis of the disease via means of molecular profiling will help better its management and treatment [4].

Proteomics, the application of high-throughput analysis of proteins, has been successfully used in the investigation of viral infections, whilst promoting identification of proteins with diagnostic or prognostic significance, as well as therapeutic targets with the ultimate goal of achieving bespoke anti-viral therapies [5]. In terms of the COVID-19 pandemic, proteomic analyses have been applied to unravel structural elements of the virus, mechanisms of pathogenesis, virus-related changes in different cell types, as well as elucidate virus–protein interactions [6]. SARS-CoV-2 proteins have been investigated using proteomics tools, and important information was generated on viral detection and potential drug targeting [7]. Additionally, the cytokine storm was shown to be a pivotal factor of mortality in patients with severe COVID-19, an outcome that can be predicted by SARS-CoV-2 Nsp3 protein analysis [8]. Further, proteomics analyses have been used to highlight alterations in molecular pathways of SARS-CoV-2 hosts (such as in nucleic acid metabolism), and such approaches can be adopted to mine for COVID-19 severity biomarkers, promote new treatment options and lead to future therapies through phosphoproteomics, glycoproteomics, and lipoproteomics [9].

Vaccination against SARS-CoV-2 is an important countermeasure to fight the ongoing pandemic. Altogether, integration of proteomics analyses has been vital in vaccine development, vaccine post authorization safety and efficacy regarding the SARS-CoV-2 wild-type or mutants [10]. Investigation of serum and/or serum by use of high-resolution techniques is elemental in vaccination analyses, especially when aiming at elucidating the heterogeneity of clinical manifestations and the complexity of immune responses to the virus [11].

Although the efficacy of anti-COVID-19 vaccination has been assessed in large clinical trials involving thousands of subjects, the underlying molecular processes and cellular mechanisms by which biological messages stimulate immune response remain poorly understood. In this frame, there exist limited studies addressing the immunological impact of anti-COVID-19 vaccination in individuals that have previously contracted SARS-CoV-2.

The aim of the study was to assess the impact of anti-SARS-CoV-2 vaccination in post -COVID-19 patients compared to non-infected individuals by analyzing serum proteomic profiles of relevant cases. Understanding how vaccines affect immune response through the study of serum proteins has huge value in increasing vaccine efficacy whilst offering new insight into the molecular mechanisms of protection from SARS-CoV-2.

## 2. Results

### 2.1. Anti-SARS-CoV-2-Induced Antibody Response

The study encompassed 252 participants aged 22–65 years old, whose anti-RBD IgG titers were monitored over a time span of six months following the second vaccination against SARS-CoV-2. Among them, 83 (33%) were males with a mean age of 47.84 years and 169 (67%) were females with a mean age of 48.00 years. Of the 252 participants, 35 had been infected and recuperated from COVID-19 infection before the first vaccine dose. The overall IgG GMC reached 980.956 AU/mL six months post-immunization, and a substantial discrepancy in antibody levels was observed in those with a history of infection (*p* < 0.001) (Table 1). The above results show that vaccination induced antibody responses in all participants included in the study. This is a finding that is in line with previously reported data on relevant clinical trials [12].

### 2.2. Global Proteomic Analysis of Post-COVID-19 Patients and Non-Infected Individuals Following Anti-SARS-CoV-2 Vaccination

**Figure 1:**
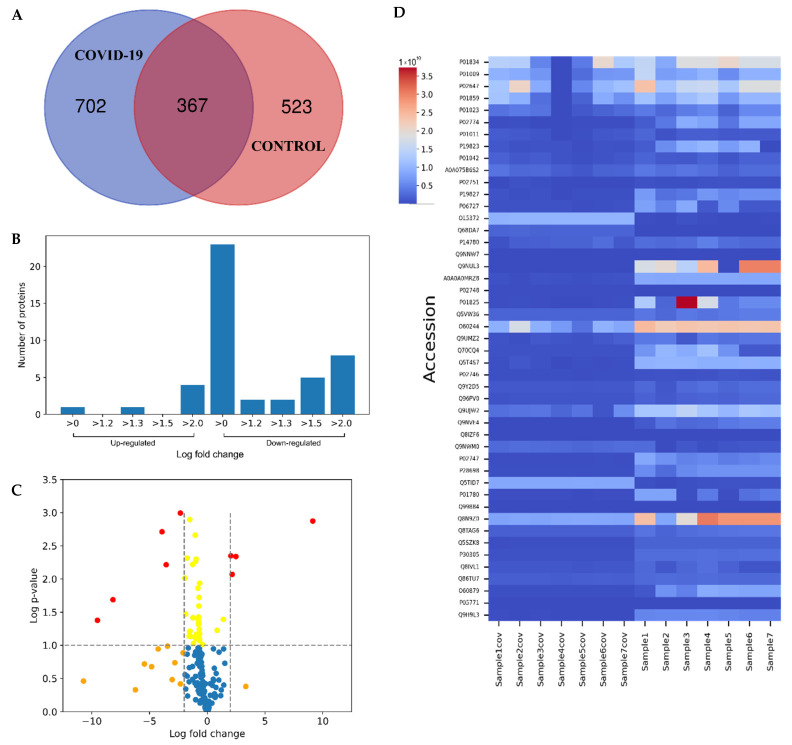
Serum proteome comparison of post-COVID-19 patients and non-infected individuals, six months post anti-SARS-COV-2 vaccination. (**A**) Venn Diagram of identified proteins in both groups. (**B**) Number of up- and down-regulated proteins with different fold changes. (**C**) Volcano plot of log2FC. (**D**) Heat map of differentially expressed proteins between the two groups.

The collected LC-MS/MS data showed detection of 5096 peptides, with 4191 (82.24%) having >2 MS/MS hits, leading to identification of 1592 serum proteins in total (Figure 1A). To determine proteins with differential expression (up- or down-regulation) between the two groups a label-free quantitative approach was undertaken on all analyzed serum samples (*n* = 20). Of the 1592 proteins, *n* = 47 were found to be consistently differentially expressed across all samples (Table 2) (Figure 1B,C). The majority of differentially expressed proteins were down-regulated (*n* = 41), and *n* = 6 were up-regulated in sera of post-COVID-19 patients vs. non-infected (Figure 1D). The full list of differentially expressed proteins with their relevant quantification data is given in Supplementary Materials 1 (S1).

### 2.3. Serum Proteomic Signatures of Anti-SARS-CoV-2 Vaccination in Post-COVID-19 Patients

**Figure 2:**
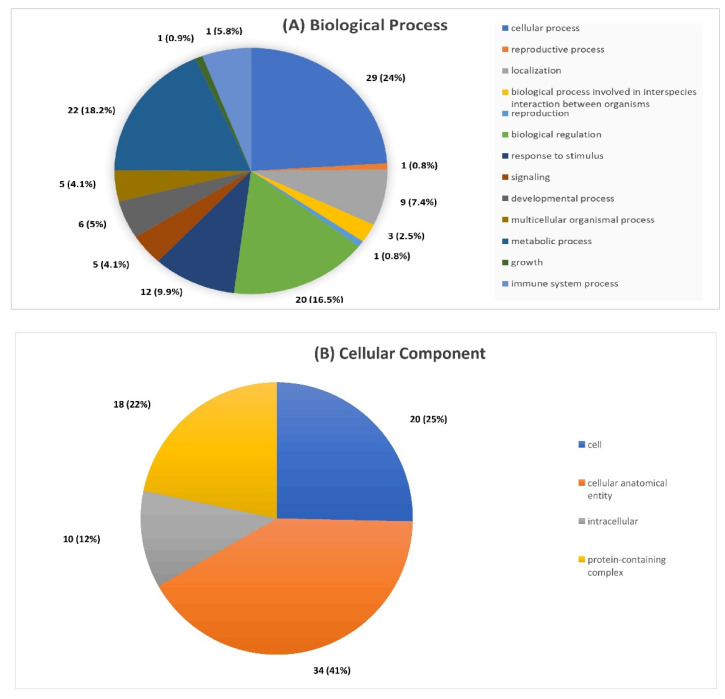

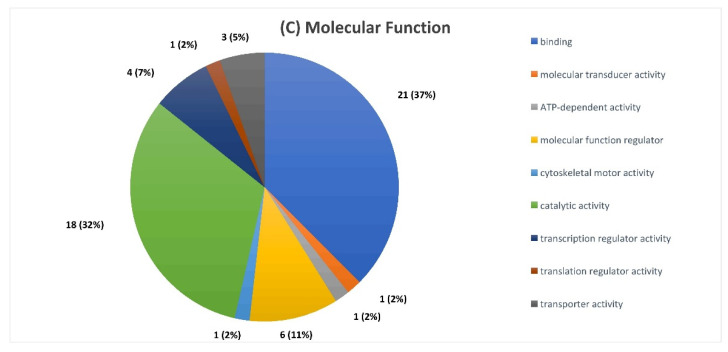
GO annotation and enrichment analysis of differentially expressed proteins between the two groups investigated.

To more accurately delve into the function and molecular significance of proteins revealed in previous steps, annotation tools were incorporated using functional databases; GO and KEGG. GO annotation and enrichment analysis of proteins with statistically significant differential expression between the two groups (*p* < 0.05) led the integration of proteins into three categories: biological process (BP), cellular component (CC) and molecular function (MF). Regarding BP, proteins were found to be mainly involved in cellular processes (24%), metabolic regulation (18%), response to stimulation (10%) and immune system processes (6%). In the CC category the majority of proteins, 41% were apportioned to the anatomical entity of the cell, 25% to the cell and 12% to the intracellular subset. Regarding the MF category, 37% of proteins had a binding function, 32% a catalytic activity and 32% were regulators of the molecular functions of the cell (Figure 2A–C).

**Figure 3:**
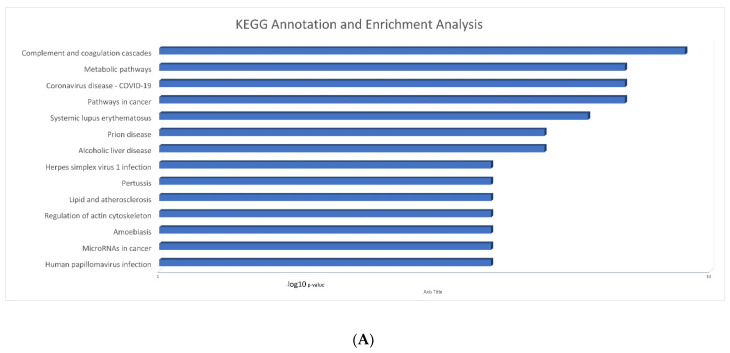

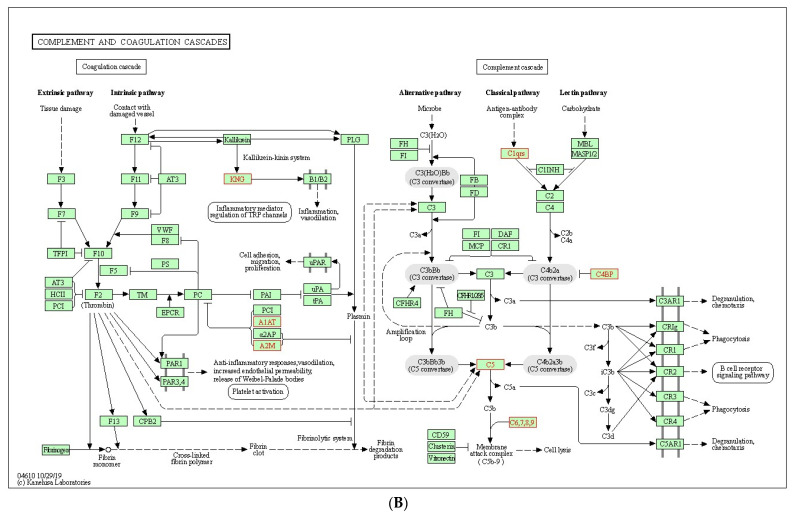
(**A**) KEGG annotation and enrichment analysis of differentially expressed proteins of the two investigated groups. (**B**) Enrichment KEGG pathway diagram showing identified proteins implicated in the complement pathway.

To elucidate the functions of differentially expressed proteins in physiological and pathological conditions as well as determine in which metabolic or signaling pathways they are involved in, a KEGG pathway analysis was undertaken. Proteins with statistically significant differential expression between the two groups (*p* < 0.05) were found to be highly enriched in processes that include all known immune-related functions, such as regulation of complement activation, humoral immune response, complement activation, etc. (Figure 3A). Interestingly, KEGG analysis revealed that the dataset of differentially expressed proteins was also enriched for COVID-19 pathways/processes, suggesting that adaptive immunity was high in vaccinated post-COVID-19 patients. The pathway with the highest KEGG enrichment was the complement and coagulation pathway (Figure 3B).

**Figure 4:**
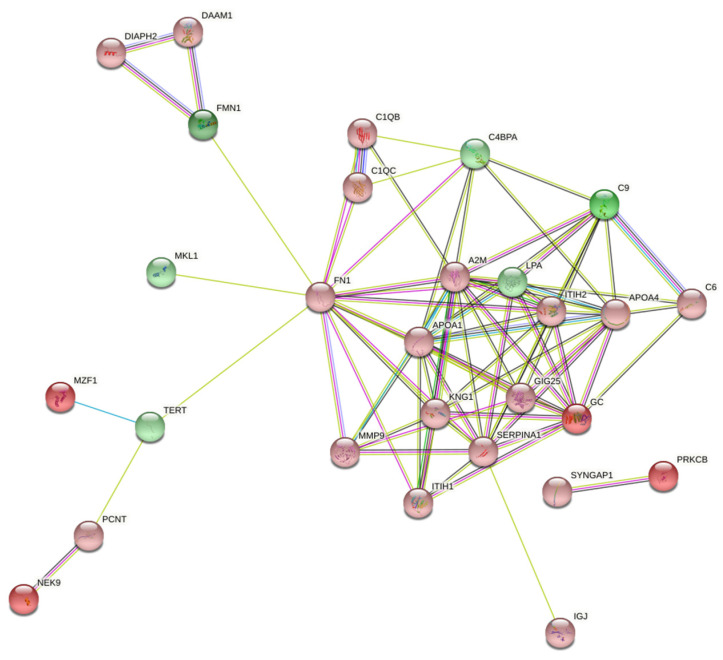
Protein interaction network of differentially expressed proteins (*p* < 0.05) involved in immune-related processes. The network includes proteins that closely interact with each other. Green color corresponds to up-regulated proteins, red color to down-regulated, and the darker the color is, the larger the value of protein up- or down-regulation.

An interaction network of differentially expressed proteins (statistical significance was set at *p* < 0.05) including molecules with the most abundant differences between the two analyzed groups was constructed using the STRING database. Each node in Figure 4 represents a protein, and each connecting line represents interactions between them. Out of the 47 differentially expressed proteins, a group of 27 were more closely related to an individual’s immune response according to the STRING database. FMN1 (Formin-1) was shown to have the maximum up-regulation value, while a high degree of up-regulation was also shown for C9 (Complement component C9). On the other hand, proteins with high down-regulation values were the MZF1 (Myeloid zinc finger 1), PRKCB (Protein kinase C beta type), NEK9 (Serine/threonine-protein kinase) and Vitamin D-binding protein.

## 3. Discussion

This study is, to our knowledge, the first to investigate the serum proteome of individuals vaccinated with an anti-SARS-CoV-2 vaccine who had been previously contracted and recuperated from COVID-19, and compare findings to the proteome of subjects with a negative history of SARS-CoV-2 infection.

Serum proteome profiling led to identification of 1592 proteins, out of which 47 presented a statistically significant differential expression in post-COVID-19 versus non-infected individuals, showcasing an alteration in relevant molecular pathways. Our results highlighted proteins involved in principally affected pathways, e.g., the complement system and coagulation cascade, in accordance with previous studies [13], as well as revealed numerous other molecules not previously connected to COVID-19 pathobiology.

The complement system lies in the front line of the host innate immune response comprising a complex of plasma proteins responsible for phagocytosis of pathogens and clearance of cellular debris. With respect to COVID-19, complement activation has been identified as a key element determining disease progression to multi-organ failure, by contributing to the hypercoagulable state, endothelial injury and other aspects characterizing severe disease course [14]. Circulating byproducts of complement activation were found significantly increased in patients with COVID-19 [15]. Our analysis showed that Complement C1q [P02746, P02746] and Complement C6 [P13671] proteins decreased in the post-COVID-19 group compared to the uninfected. C1q is part of the inactive C1 complex of the classical pathway, while C6 is a pore-like structure belonging to the membrane attack complex formed in the latest stages of the complement cascade. A study reported up-regulation of C1q in patients with mild-to-critical COVID-19, compared to healthy controls, while no difference was detected between patients with mild and critical disease course [16]. Our study design differed from the latter with regard to the vaccination status of included individuals, which may provide an explanation as to why the aforementioned molecules were found downregulated.

Our results further showed that the complement components C4b-binding protein and its alpha chain [P04003], as well as C9 [P02748] were elevated in the post-COVID-19 patient group compared to the uninfected. Consistent evidence was provided by a study reporting C4 elevation in patients with possible SARS-CoV-2 infection held in the Emergency Department [17]. Similarly, another study on patients with severe COVID-19 found elevated deposits of C5a to C9 in biopsies of skin and lung tissue [18]. Notably, all patients participating in earlier studies were unvaccinated, while all the participants in our study were vaccinated, suggesting that vaccination does not eliminate up-regulation of complement components.

Formin-like 1 (FMN1) a member of the formin family is expressed in T cells, involved in macrophage phagocytosis, migration, and CD8 T-cell cytotoxicity. Up-regulation of FMN1 in T cells has been observed in the context of autoimmunity, while impaired expression of FMN1 has been found to hinder the induction of autoimmune diseases [19,20]. The coding gene of FMN1 was found to be highly expressed in lung cell types of COVID-19 patients compared to healthy controls [21]. Of note, the 17q21.31 locus, which derives from highly pleiotropic inversion polymorphism and has been reported in the COVID-19 Host Genetics Initiative, was significantly correlated with greater disease severity [22]. Our study revealed that serum levels of FMN1 [Q68DA7] were significantly elevated in the post-COVID-19 group compared to the uninfected. Given the aforementioned properties of this molecule, this finding confirms an increased T cell activity in the former group.

Zinc is a vital element for the proper function of our immune system against infections acting as an immunomodulator and inhibiting viral replication [23]. Studies have highlighted the role of zinc in COVID-19 as being the main ion that binds to SARS-CoV-2 through several zinc-binding sites recognized through proteomic analysis of the virus [24]. These studies have evidenced the binary effect of zinc on SARS-CoV-2, as its binding can prove both fundamental for viral reproduction as well as toxic. In our study, zinc-transporter-10 [Q6XR72] and myeloid zinc finger 1 [P28698], showcasing a structural connection between viral and eukaryotic binding sites, were found significantly diminished in the post-COVID-19 group compared to controls, indicating the capacity of SARS-CoV-2 to compete with host proteins for zinc binding. This finding can be attributed to the knowledge that zinc transporters and other accessory agents are often recruited to serve viral replication, accounting for the most part of human metalloproteome involvement in the SARS-CoV-2 infection [25]. Moreover, proteins containing zinc fingers have been identified as pivotal for disease progression, due to their engagement both in host antiviral mechanisms and in the viral reproduction cycle, thus explaining their drop in previously infected individuals [26].

Mucins are a family of high-molecular weight proteins located on the apical surfaces of airway epithelia, playing a major role in the host front-line protection of the respiratory tract by forming a protective mucous barrier against invading pathogens. The gel-like mucin layer formed in the airway epithelia has been accused of enhancing membrane fusion and invasion of SARS-CoV-2, while high viral load and mucin hyperproduction constraints proper air flow and causes ciliary dyskinesia, impeding airway clearance further aggravating the respiratory function [27]. Mucin hypersecretion has been observed as a result of the cytokine storm and has been held responsible for activating inflammatory processes that further exacerbate disease severity, while the mucin barrier also hinders therapeutic delivery to the respiratory tract [28]. In line with the above, our findings reported a drop in mucin-16 [Q8WXI7] levels in the COVID-19 group compared to uninfected controls. A study conducting a transcriptome-wide correlation aiming to unveil the impact of gene expression on COVID-19 course revealed that up-regulated expression of MUC1 was associated with critical disease [29], while MUC5AC and MUC5B mucins have also been found to be elevated in severe COVID-19 [30,31]. Our results attest to a forefront role of this molecule on COVID-19 pathobiology.

Studies have proven an association of vitamin D status with COVID-19 severity, reporting that diminished serum levels of this molecule were correlated with a poor disease prognosis including a higher risk of ICU admission and mortality [32]. Another study suggested that certain vitamin D binding protein (VTDB) polymorphisms lie beneath this observation [33]. VTDB is the primary transporter of the main vitamin D metabolites that are protein bound, serving as a hormonal reservoir that defines the median plasma concentration of these metabolites. In the context of acute respiratory distress syndrome (ARDS), VTDB levels have been found to be significantly decreased compared to healthy controls, suggesting that several VTDB polymorphisms aggravate COVID-19 severity [34]. Our study detected a significant decrease in VTDB [P02774] levels in the post-COVID-19 patient group compared to controls, which is a quite anticipated finding based on evidence reported in the literature.

Fibronectin is an extracellular matrix glycoprotein essential for cell adhesion, chemotaxis and differentiation, acting as a link between inflammatory cells and collagen and regulating the innate immune response [35]. An observational study performed in critically ill COVID-19 patients highlighted the role of fibronectin as a marker of disease severity, displaying a significant elevation in non-survivors [36]. Another study designed a recombinant protein containing fibronectin type III domain (FN3), which in synergy with other molecules formed a potential long-acting antiviral drug effective against a broad spectrum of SARS-CoV-2 variants and other coronaviruses [37]. Other investigators have also confirmed the role of FN3 in the development of anti-inflammatory drug candidates targeting the cytokine storm generated by SARS-CoV-2 [38]. Our study revealed a down-regulation of fibronectin [P02751], portraying a possible effect of anti-SARS-CoV-2 vaccination, whilst underlying how importantly the SARS-CoV-2 virus alters the expression of extracellular matrix proteins, including fibronectin.

Coiled coil (CC) dimer-forming peptides comprise a distinct pattern found in a variety of proteins characterized by a defined set of assembly and affinity rules, rendering it highly eligible for the design of scaffolds and functional proteins. A recent study developed an assay harnessing these properties of CC pairs to detect membrane fusion caused by the interaction of the SARS-CoV-2 spike protein with angiotensin converting enzyme 2 (ACE2) receptors [39]. Our findings attested to a marked down-regulation of the coiled coil domain B7 containing protein [Q5TID7] in post-COVID-19 patients compared to controls. This correlation, made for the first time, reveals a new role of this molecule with regard to COVID-19 frame of affected proteins.

NEKs include eleven proteins engaged in a variety of cellular functions such as cell trafficking and highly associated with p53, a tumor suppressor gene known to display a protective effect on the lung microvascular network. In particular, NEK9 activates Ras homolog family member A (RhoA) promoting phosphorylation of the myosin light chain 2. In an in vivo study employing a murine sepsis model, the role of NEKs in endothelial barrier dysfunction, a state characterizing ARDS, was examined [40]. This study revealed the involvement of NEKs, including NEK9, in inflammatory pathways, reporting their up-regulation in sepsis and highlighting their role as potential therapeutic targets in sepsis-derived ARDS. The significant down-regulation of NEK9 [Q8TD19] observed in our study in the previously infected group lies well in line with the fact that none of our study participants underwent a severe disease course, indicating a protective residue of infection-acquired immunity.

Inhibition of protein kinases C alpha and beta promotes erythrocyte survival, while celerythrine, a specific inhibitor of these kinases has been found to counteract the eryptosis caused by antiviral drugs recruited against SARS-CoV-2. Moreover, this novel coronavirus has been found to trigger phosphorylation of host kinases, among which protein kinase C, whose levels have been found elevated in SARS-CoV-2 infected cells [41]. A significant downregulation of protein kinase C beta [P05771] in the COVID-19 group compared to controls was evidenced in our study, a finding that warrants further investigation.

## 4. Materials and Methods

### 4.1. Patient Samples and Experimental Design

**Figure 5:**
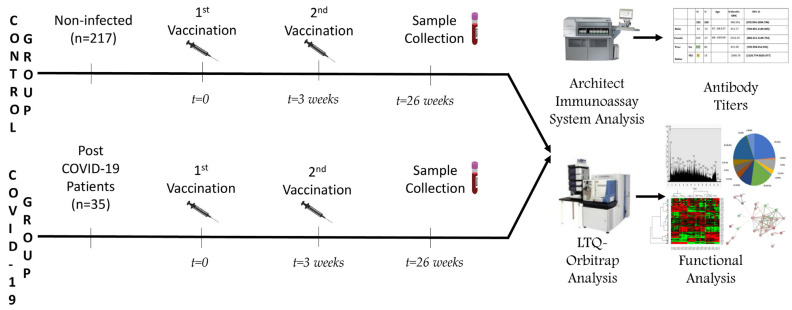
Study overview. Blood samples from *n* = 252 individuals were collected following anti-SARS-CoV-2 vaccination. Of these *n* = 35 samples were from post-COVID-19 patients. All samples underwent COVID-19-specific IgG quantitative determination by the SARS-CoV-2 IgG II Quant assay. In parallel quantitative LC-MS/MS analyses were performed and the identified proteins were analysed using the GO (Gene Ontology) database and KEGG (Kyoto Encyclopedia of Genes) database.

Blood samples of healthcare workers of the “G. Gennimatas” General Hospital of Thessaloniki who received the BNT162b2 mRNA COVID-19 vaccine (Pfizer/BioNtech) (Pfizer, New York, NY, USA) (*n* = 252), were collected 6 months post-immunization with the second vaccine dose, from 1/4/2021 to 8/31/2021. Blood sampling was carried out under the supervision of the staff at the Microbiological Laboratory of “G. Gennimatas” Hospital, after obtaining signed informed consent of the participants. The samples were centrifuged and, following the measurement of anti-SARS-CoV-2 antibodies in the serum of the study participants, the samples were frozen (−80 °C) until subsequent proteomic analysis. Of the total study population, a subset of *n* = 35 individuals had already been infected and recuperated from COVID-19 before the first vaccination dose. The study workflow is depicted in Figure 5. Immunization took place in the two vaccination centers of “G. Gennimatas” General Hospital of Thessaloniki from 8 February 2021 up to 24 April 2021. Written informed consent was obtained from all participants. Approval of the study protocol was obtained by the Ethics Committee of the Scientific Council of the G. Gennimatas General Hospital (protocol number: 1/13.1.2021), in accordance with the Declaration of Helsinki and the International Conference on Harmonization for Good Clinical Practice.

### 4.2. COVID-19-Specific IgG Quantitative Determination

In all study participants, quantification of RBD-specific IgG antibodies against SARS-CoV-2 was performed in serum-derived samples. Titers of total RBD-specific IgGs against SARS-CoV-2 were determined using the SARS-CoV-2 IgG II Quant assay on the ARCHITECT System (SARS-CoV-2 IgG II Quant, Abbott Sligo, Ireland). The SARS-CoV-2 IgG II Quant assay is a chemiluminescent microparticle immunoassay (CMIA) used for the qualitative and quantitative determination of IgG antibodies to SARS-CoV-2 in human serum and plasma on the Alinity and ARCHITECT i Systems. This assay is to be used in evaluating the immune status of infected individuals and monitor antibody response in individuals that have received the COVID-19 vaccine, by quantitatively measuring IgG antibodies against the spike receptor-binding domain (RBD) of SARS-CoV-2. A value of anti-S IgG antibodies >50 AUs was considered the cut-off for positivity (according to the manufacturer’s instructions). The geometric mean concentration (GMC) and respective 95% confidence intervals were calculated based on the recorded antibody concentration values.

### 4.3. Proteomic Investigation

Of the collected blood samples, twenty (*n* = 10 from post-COVID-19 patients and *n* = 10 belonging to individuals with no prior infection) were selected for further processing.

Following thawing, samples were centrifuged, and serum was collected for proteomic analysis. Protein concentration was determined by the Bradford assay, and 20 μg were dissolved in lysis buffer [4% *w/w* SDS, 0.1 M Tris-HCL, 0.1 M dithioerythritol (DTE) pH = 7.6]. Denaturated samples were transferred to Amicons Ultra 0.5 Centrifugal Filter Devices (Merck, Darmstadt, Germany), 8 M urea solution was added and samples were centrifuged for 15 min at 13.000 rpm, as previously described [42]. An alkylation step followed by addition of iodoacetamide (0.05 M iodoacetamide in 8 M urea, 0.1 M Tris-HCL pH = 8.5) and filter units were left in the dark for 20 min. Lastly, a trypsin solution was added (enzyme to protein ratio: 1:100), and digestion took place overnight.

For LC-MS/MS analysis, peptide separation was performed by a reversed-phase analytical C-18 column (75 μm × 50 cm; 100 Å, 2-μm-bead-packed Acclaim PepMap RSLC, Thermo Scientific), using an Ultimate-3000 system (Dionex, Thermo Scientific, Bremen, Germany) interfaced to an LTQ-Velos Orbitrap Elite mass spectrometer (Thermo Scientific, Waltham, MA, USA). Initially, 20 ng were loaded on a C-18 pre-column at a 5 μL/min flow rate in phase A (0.1% formic acid (*v*/*v*) in 99.9% HPLC grade water (Thermo Fisher Scientific, Foster City, CA, USA). The elution time lasted 240 min and had a gradient of 2–35% phase B (99.9% acetonitrile, 0.1% formic acid) at a 300 nL/min flow rate.

The collected MS/MS spectra files were processed in the Proteome Discoverer protein bioinformatics platform (version 1.4.0.388) (Thermo Scientific). Data were searched with the SEQUEST engine against the *Homo sapiens* *.fasta database of Uniprot (version 6/2022). The Proteome Discoverer parameters used were: two maximum missed cleavage points for trypsin; oxidation of methionine as a variable modification; 5 ppm peptide mass tolerance; and 0.01 ppm fragment ion tolerance. A percolator based on q-values at a 0.01% false discovery rate (FDR) validated the peptide spectral matches, and extra peptide filtering was performed with an Xcorr versus peptide charge values (percolator maximum Delta Cn was set at 0.05).

### 4.4. Data Analysis

Protein quantification was performed using the T-test. Protein significance was evaluated only for proteins present in the entity of analyzed samples. Differentially expressed proteins were defined as proteins with statistically significant differences in quantification between the two analyzed groups (*p* < 0.05, |log2FC| > 1 (ratio > 1 or ratio < 1 [fold change, FC]).

The identified proteins were annotated with common functional databases, including the GO (Gene Ontology) database and KEGG (Kyoto Encyclopedia of Genes and Genomes) database. The quantification of the identified proteins was followed by screening and clustering of expression patterns of the identified proteins. Functional analyses, such as GO and KEGG functional enrichment analysis and interaction network analysis, were performed on all differential expressed proteins.

## 5. Conclusions

Overall, our findings contribute novel insights into the mechanisms of immune response induced by anti-SARS-CoV-2 vaccination, whilst identifying genomic and proteomic signatures that reflect the immune status of vaccine recipients. The significant pathways that were found affected in our study highlight the crucial underlying mechanisms involved in the humoral immune response elicited by vaccination, that are recruited for our protection against COVID-19. Our molecular investigation shed light on the fine points of the adaptive immune response of vaccinees, paving the way for future vaccinomic studies that will further elucidate the leading role of vaccination as our primary prophylactic measure against the ongoing pandemic.

## Figures and Tables

**Table 1:** Results of COVID-19-specific IgG quantitative determination, and demographic data of study participants.

		N	%	Age	6 Months GMC	95% CI
		252	100	22–65	980.956	(878.954–1094.794)
Male		83	33	47.84337	912.77	(730.801–1140.045)
Female		169	67	48.00599	1016.29	(898.312–1149.754)
Prior Status	No	217	86		825.98	(745.958–914.591)
	YES	35	14		2848.78	(2120.774–3826.677)

**Table 2:** Differential protein analysis.

Num. of Total Quant.	Regulation Type	Fold-Change				
		**>0**	**>1.2**	**>1.3**	**>1.5**	**>2.0**
**47**	Up-regulated	1	0	1	0	4
	Down-regulated	23	2	2	5	8

## Data Availability

Data are available by the corresponding author, upon reasonable request.

## Supplementary Materials

The supporting information can be downloaded at: https://www.mdpi.com/article/10.3390/ijms231710153/s1.
