# Supplementary material for: Serum Proteome Signatures of Anti-SARS-CoV-2 Vaccinated Healthcare Workers in Greece Associated with Their Prior Infection Status
Source: Int J Mol Sci. 2022 Sep 5;23(17):10153. doi: 10.3390/ijms231710153 (PMC9456361; doi:10.3390/ijms231710153)
Supplement: Supplementary file 1 [file ijms-23-10153-s001.zip › ijms-1870902-supplementary.pdf]

Supplementary Materials 1 (S1)

Table S1. Information on proteins differentially expressed in post-Covid-19 patients compared to non-infected individuals following anti-SARS-COV-2 vaccination.

Accession	Description	Mean Covid	Mean Noncovid	Log2FoldChange	U/D
P01834	Immunoglobulin kappa constant OS=Homo sapiens OX=9606 GN=IGKC PE=1 SV=2 - [IGKC_HUMAN]	9955715381	16657596825	-0,742583388	DOWN
P01009	Alpha-1-antitrypsin OS=Homo sapiens OX=9606 GN=SERPINA1 PE=1 SV=3 - [A1AT_HUMAN]	5919086323	9363700856	-0,661704348	DOWN
P02647	Apolipoprotein A-I OS=Homo sapiens OX=9606 GN=APOA1 PE=1 SV=1 - [APOA1_HUMAN]	9828250538	16627630261	-0,758576033	DOWN
P01859	Immunoglobulin heavy constant gamma 2 OS=Homo sapiens OX=9606 GN=IGHG2 PE=1 SV=2 - [IGHG2_HUMAN]	5880637233	10077059314	-0,777030293	DOWN
P01023	Alpha-2-macroglobulin OS=Homo sapiens OX=9606 GN=A2M PE=1 SV=3 - [A2MG_HUMAN]	2368480123	4031745501	-0,767443009	DOWN
P02774	Vitamin D-binding protein OS=Homo sapiens OX=9606 GN=GC PE=1 SV=2 - [VTDB_HUMAN]	157989822,1	794156814,7	-2,329592289	DOWN
P01011	Alpha-1-antichymotrypsin OS=Homo sapiens OX=9606 GN=SERPINA3 PE=1 SV=2 - [AACT_HUMAN]	837358446	1335160036	-0,673095447	DOWN
P19823	Inter-alpha-trypsin inhibitor heavy chain H2 OS=Homo sapiens OX=9606 GN=ITIH2 PE=1 SV=2 - [ITIH2_HUMAN]	567426654,5	920754250	-0,698382229	DOWN
P01042	Kininogen-1 OS=Homo sapiens OX=9606 GN=KNG1 PE=1 SV=2 - [KNG1_HUMAN]	951363764,4	1574457549	-0,726785878	DOWN
A0A075B6S2	Immunoglobulin kappa variable 2D-29 OS=Homo sapiens OX=9606 GN=IGKV2D-29 PE=3 SV=1 - [KVD29_HUMAN]	2233959663	2959455057	-0,405728412	DOWN
P02751	Fibronectin OS=Homo sapiens OX=9606 GN=FN1 PE=1 SV=5 - [FINC_HUMAN]	272654808,6	476322431,5	-0,804862892	DOWN
P19827	Inter-alpha-trypsin inhibitor heavy chain H1 OS=Homo sapiens OX=9606 GN=ITIH1 PE=1 SV=3 - [ITIH1_HUMAN]	340963148	538451704,6	-0,659201133	DOWN

P06727	Apolipoprotein A-IV OS=Homo sapiens OX=9606 GN=APOA4 PE=1 SV=4 - [APOA4_HUMAN]	432714368,5	865340716,2	-0,999853259	DOWN
Q86WZ6	Zinc finger protein 227 OS=Homo sapiens OX=9606 GN=ZNF227 PE=1 SV=1 - [ZN227_HUMAN]	8700050155	10565817104	-0,280308719	DOWN
Q9Y673	Dolichyl-phosphate beta-glucosyltransferase OS=Homo sapiens OX=9606 GN=ALG5 PE=1 SV=1 - [ALG5_HUMAN]	14372710840	23326469190	-0,698635756	DOWN
O15372	Eukaryotic translation initiation factor 3 subunit H OS=Homo sapiens OX=9606 GN=EIF3H PE=1 SV=1 - [EIF3H_HUMAN]	976075175	237138826,3	2,041260373	UP
Q68DA7	Formin-1 OS=Homo sapiens OX=9606 GN=FMN1 PE=1 SV=3 - [FMN1_HUMAN]	2099576388	3724833,266	9,138706715	UP
P14780	Matrix metalloproteinase-9 OS=Homo sapiens OX=9606 GN=MMP9 PE=1 SV=3 - [MMP9_HUMAN]	1516284714	2897969765	-0,934501868	DOWN
Q9H1P3	Oxysterol-binding protein-related protein 2 OS=Homo sapiens OX=9606 GN=OSBPL2 PE=1 SV=1 - [OSBL2_HUMAN]	922164858	3937288080	-2,094105681	DOWN
Q9NNW7	Thioredoxin reductase 2, mitochondrial OS=Homo sapiens OX=9606 GN=TXNRD2 PE=1 SV=3 - [TRXR2_HUMAN]	1076902,516	3194821,185	-1,568847527	DOWN
Q9NUL3	Double-stranded RNA-binding protein Staufen homolog 2 OS=Homo sapiens OX=9606 GN=STAU2 PE=1 SV=2 - [STAU2_HUMAN]	27157736,51	19808501699	-9,510540803	DOWN
P01860	Immunoglobulin heavy constant gamma 3 OS=Homo sapiens OX=9606 GN=IGHG3 PE=1 SV=2 - [IGHG3_HUMAN]	8001190179	5497596321	0,541413641	UP
P01591	Immunoglobulin J chain OS=Homo sapiens OX=9606 GN=JCHAIN PE=1 SV=4 - [IGJ_HUMAN]	297770793,5	391657506	-0,395390351	DOWN
P04003	C4b-binding protein alpha chain OS=Homo sapiens OX=9606 GN=C4BPA PE=1 SV=2 - [C4BPA_HUMAN]	338903632,4	167075967,6	1,02037087	UP
A0A0A0MRZ8	Immunoglobulin kappa variable 3D-11 OS=Homo sapiens OX=9606 GN=IGKV3D-11 PE=3 SV=6 - [KVD11_HUMAN]	512187014,8	835925170	-0,706703124	DOWN
P02748	Complement component C9 OS=Homo sapiens OX=9606 GN=C9 PE=1 SV=2 - [CO9_HUMAN]	193718498,1	34781156,56	2,477583911	UP

P01825	Immunoglobulin heavy variable 4-59 OS=Homo sapiens OX=9606 GN=IGHV4-59 PE=1 SV=2 - [HV459_HUMAN]	207519993,6	417398641	-1,008175563	DOWN
Q5VVW2	GTPase-activating Rap/Ran-GAP domain-like protein 3 OS=Homo sapiens OX=9606 GN=GARNL3 PE=2 SV=2 - [GARL3_HUMAN]	527518377	441345564,3	0,257312655	UP
Q8N3K9	Cardiomyopathy-associated protein 5 OS=Homo sapiens OX=9606 GN=CMYA5 PE=1 SV=3 - [CMYA5_HUMAN]	121089787,8	144710313,2	-0,257090544	DOWN
Q9Y4D1	Disheveled-associated activator of morphogenesis 1 OS=Homo sapiens OX=9606 GN=DAAM1 PE=1 SV=2 - [DAAM1_HUMAN]	1654473554	2167236422	-0,389484314	DOWN
Q5VW36	Focadhesin OS=Homo sapiens OX=9606 GN=FOCAD PE=1 SV=1 - [FOCAD_HUMAN]	2163924304	3484663181	-0,687369182	DOWN
O60244	Mediator of RNA polymerase II transcription subunit 14 OS=Homo sapiens OX=9606 GN=MED14 PE=1 SV=2 - [MED14_HUMAN]	9754629745	22366385024	-1,19717308	DOWN
Q9UMZ2	Synergin gamma OS=Homo sapiens OX=9606 GN=SYNRG PE=1 SV=2 - [SYNRG_HUMAN]	875086647,3	3337151673	-1,93111948	DOWN
Q70CQ4	Ubiquitin carboxyl-terminal hydrolase 31 OS=Homo sapiens OX=9606 GN=USP31 PE=2 SV=2 - [UBP31_HUMAN]	533825487,8	1093958420	-1,03511781	DOWN
Q5T4S7	E3 ubiquitin-protein ligase UBR4 OS=Homo sapiens OX=9606 GN=UBR4 PE=1 SV=1 - [UBR4_HUMAN]	589704833,4	42764383164	-6,180272902	DOWN
Q86YA3	Protein ZGRF1 OS=Homo sapiens OX=9606 GN=ZGRF1 PE=1 SV=3 - [ZGRF1_HUMAN]	490995503,7	539346772	-0,135503336	DOWN
P02746	Complement C1q subcomponent subunit B OS=Homo sapiens OX=9606 GN=C1QB PE=1 SV=3 - [C1QB_HUMAN]	344920849,3	570159597,7	-0,725100474	DOWN
Q9Y2D5	A-kinase anchor protein 2 OS=Homo sapiens OX=9606 GN=AKAP2 PE=1 SV=3 - [AKAP2_HUMAN]	1007269294	2408178442	-1,257492857	DOWN
Q969V6	Myocardin-related transcription factor A OS=Homo sapiens OX=9606 GN=MRTFA PE=1 SV=1 - [MRTFA_HUMAN]	4826383266	3156184865	0,612760776	UP
Q96PV0	Ras/Rap GTPase-activating protein SynGAP OS=Homo sapiens OX=9606 GN=SYNGAP1 PE=1 SV=4 - [SYGP1_HUMAN]	751141714	2006822322	-1,417755866	DOWN

Q9UJW2	Tubulointerstitial nephritis antigen OS=Homo sapiens OX=9606 GN=TINAG PE=2 SV=3 - [TINAG_HUMAN]	3418594692	12482085637	-1,868383723	DOWN
Q9NVE4	Coiled-coil domain-containing protein 87 OS=Homo sapiens OX=9606 GN=CCDC87 PE=1 SV=2 - [CCD87_HUMAN]	11504975	3307765827	-8,167455444	DOWN
Q8IZF6	Adhesion G-protein coupled receptor G4 OS=Homo sapiens OX=9606 GN=ADGRG4 PE=2 SV=2 - [AGRG4_HUMAN]	10577082,75	4014398,021	1,397686205	UP
Q9NWM0	Spermine oxidase OS=Homo sapiens OX=9606 GN=SMOX PE=1 SV=1 - [SMOX_HUMAN]	2299610580	1276149431	0,849592302	UP
P02747	Complement C1q subcomponent subunit C OS=Homo sapiens OX=9606 GN=C1QC PE=1 SV=3 - [C1QC_HUMAN]	238391296,5	564639144,9	-1,243997585	DOWN
Q86WB7	Protein unc-93 homolog A OS=Homo sapiens OX=9606 GN=UNC93A PE=1 SV=1 - [UN93A_HUMAN]	990318549,9	2076597412	-1,068256981	DOWN
P28698	Myeloid zinc finger 1 OS=Homo sapiens OX=9606 GN=MZF1 PE=1 SV=3 - [MZF1_HUMAN]	362886615,3	5486207184	-3,918218355	DOWN
Q5TID7	Coiled-coil domain-containing protein 181 OS=Homo sapiens OX=9606 GN=CCDC181 PE=2 SV=1 - [CC181_HUMAN]	8537651775	1913065231	2,157953254	UP
P01780	Immunoglobulin heavy variable 3-7 OS=Homo sapiens OX=9606 GN=IGHV3-7 PE=1 SV=2 - [HV307_HUMAN]	184049995,5	534248055,8	-1,537412038	DOWN
Q99884	Sodium-dependent proline transporter OS=Homo sapiens OX=9606 GN=SLC6A7 PE=2 SV=2 - [SC6A7_HUMAN]	34045027,03	62021583,97	-0,865326293	DOWN
P08519	Apolipoprotein(a) OS=Homo sapiens OX=9606 GN=LPA PE=1 SV=1 - [APOA_HUMAN]	3294171584	1934674112	0,767825132	UP
P01624	Immunoglobulin kappa variable 3-15 OS=Homo sapiens OX=9606 GN=IGKV3-15 PE=1 SV=2 - [KV315_HUMAN]	274535888	496399309	-0,854506353	DOWN
Q9H3Z4	DnaJ homolog subfamily C member 5 OS=Homo sapiens OX=9606 GN=DNAJC5 PE=1 SV=1 - [DNJC5_HUMAN]	184833310,3	3528472689	-4,254747159	DOWN
Q8N9Z0	Zinc finger protein 610 OS=Homo sapiens OX=9606 GN=ZNF610 PE=2 SV=2 - [ZN610_HUMAN]	8537651775	24013784188	-1,491951541	DOWN

O15265	Ataxin-7 OS=Homo sapiens OX=9606 GN=ATXN7 PE=1 SV=1 - [ATX7_HUMAN]	3239989049	3115528708	0,056511926	UP
Q8TAG6	Vexin OS=Homo sapiens OX=9606 GN=VXN PE=1 SV=2 - [VEXIN_HUMAN]	1638188429	3200848010	-0,966352864	DOWN
Q5SZK8	FRAS1-related extracellular matrix protein 2 OS=Homo sapiens OX=9606 GN=FREM2 PE=1 SV=2 - [FREM2_HUMAN]	306833685	1981068372	-2,690749893	DOWN
P30305	M-phase inducer phosphatase 2 OS=Homo sapiens OX=9606 GN=CDC25B PE=1 SV=2 - [MPIP2_HUMAN]	447841745	2942764603	-2,716111225	DOWN
Q8IVL1	Neuron navigator 2 OS=Homo sapiens OX=9606 GN=NAV2 PE=1 SV=3 - [NAV2_HUMAN]	855901959,7	1706943096	-0,995897509	DOWN
Q8TD19	Serine/threonine-protein kinase Nek9 OS=Homo sapiens OX=9606 GN=NEK9 PE=1 SV=2 - [NEK9_HUMAN]	7569551,25	82335083,77	-3,443227628	DOWN
O95613	Pericentrin OS=Homo sapiens OX=9606 GN=PCNT PE=1 SV=4 - [PCNT_HUMAN]	571735492,3	940097420,5	-0,717462415	DOWN
Q86TU7	Actin-histidine N-methyltransferase OS=Homo sapiens OX=9606 GN=SETD3 PE=1 SV=1 - [SETD3_HUMAN]	1590633326	2495817131	-0,649910929	DOWN
Q6XR72	Zinc transporter 10 OS=Homo sapiens OX=9606 GN=SLC30A10 PE=1 SV=2 - [ZNT10_HUMAN]	305810604,5	13314204230	-5,444183958	DOWN
Q8WXI7	Mucin-16 OS=Homo sapiens OX=9606 GN=MUC16 PE=1 SV=3 - [MUC16_HUMAN]	1368552552	11208683777	-3,033894134	DOWN
O60879	Protein diaphanous homolog 2 OS=Homo sapiens OX=9606 GN=DIAPH2 PE=1 SV=1 - [DIAP2_HUMAN]	311011164,5	882571794	-1,504747271	DOWN
P05771	Protein kinase C beta type OS=Homo sapiens OX=9606 GN=PRKCB PE=1 SV=4 - [KPCB_HUMAN]	1923723,425	22677236	-3,559271507	DOWN
Q8IY33	MICAL-like protein 2 OS=Homo sapiens OX=9606 GN=MICALL2 PE=1 SV=1 - [MILK2_HUMAN]		228961413,7		
Q58EX2	Protein sidekick-2 OS=Homo sapiens OX=9606 GN=SDK2 PE=1 SV=3 - [SDK2_HUMAN]	172961938,1	330178117,7	-0,932789916	DOWN
P13671	Complement component C6 OS=Homo sapiens OX=9606 GN=C6 PE=1 SV=3 - [CO6_HUMAN]	73782519,5	148701642,3	-1,011069621	DOWN
O14746	Telomerase reverse transcriptase OS=Homo sapiens OX=9606	728533150,5	508225435,8	0,519526039	UP

	GN=TERT PE=1 SV=1 - [TERT_HUMAN]				
Q9H9L3	Interferon-stimulated 20 kDa exonuclease-like 2 OS=Homo sapiens OX=9606 GN=ISG20L2 PE=1 SV=1 - [I20L2_HUMAN]	144615889	484920563,3	-1,745522363	DOWN
